# *Mycobacterium tuberculosis* Beijing Type Mutation Frequency

**DOI:** 10.3201/eid1903.121001

**Published:** 2013-03

**Authors:** Jim Werngren

**Affiliations:** Author affiliation: Swedish Institute for Communicable Disease Control, Solna, Sweden

**Keywords:** Mycobacterium tuberculosis, Beijing type, drug resistance, mutation frequency, tuberculosis and other mycobacteria

**To the Editor:** A striking finding in the study by de Steenwinkel et al. (*1*) is the high frequency of mutation to rifampin resistance by 2 *Mycobacterium tuberculosis* Beijing strains, which might play a role in the association between the Beijing strains and multidrug-resistant tuberculosis. Earlier reported frequency of mutation to rifampin resistance by *M. tuberculosis* has been 10^−8^ CFU (*2**,**3*), including the Beijing genotype (*3**,**4*). Of note, the Beijing 2002–1585 strain, for which frequency of mutation to rifampin resistance is 10^−3^ CFU (1 mutant/1,000 CFU), showed a moderate frequency of 10^−8^ CFU in another study (*4*). We think that a mutation frequency increase of 100,000× is remarkably high. In contrast, rifampin-resistant mutants of the Beijing 1585 strain did not emerge in low-density cultures (5 × 10^5^ CFU/mL) used for time-kill kinetics experiments, although frequency of mutation to rifampin resistance was determined to be 10^−3^ CFU.

Mutation frequency is determined by fluctuation assays. To exclude preexisting mutants, which would bias the mutation frequency by so-called jackpots, a series of low-inoculum cultures is typically used (*5*). However, for unknown reasons, de Steenwinkel et al. used only 1 high-density culture of 10^10^ CFU of each strain to determine mutation frequency. This strategy is not recommended because mutations can occur early or late, resulting in substantial mutation frequency fluctuation between test episodes. A strain with known mutation rates should preferably be included to rule out possible technical errors.

We propose the following explanations for the remarkable results: 1) the rifampin concentration for selecting mutants might have been too low, enabling growth of some colonies of drug-susceptible bacteria; 2) rifampin mutants arose early or preexisted in the cultivation of Beijing strains 1585 and 1607, producing jackpots; or 3) the 2 Beijing isolates might contain rifampin-resistant subpopulations (heteroresistance). The capacity of the Beijing strain to develop and, especially, transmit multidrug-resistant tuberculosis remains to be further analyzed.
